# C-Met targeted fluorescence molecular endoscopy in Barrett's esophagus patients and identification of outcome parameters for phase-I studies

**DOI:** 10.7150/thno.42224

**Published:** 2020-04-06

**Authors:** Steven J. de Jongh, Floris J. Voskuil, Iris Schmidt, Arend Karrenbeld, Gursah Kats-Ugurlu, Gert Jan Meersma, Jessie Westerhof, Max J.H. Witjes, Gooitzen M. van Dam, Dominic J. Robinson, Wouter B. Nagengast

**Affiliations:** 1 Department of Gastroenterology and Hepatology, University Medical Center Groningen, Groningen, The Netherlands;; 2 Department of Oral and Maxillofacial Surgery, University Medical Center Groningen, Groningen, The Netherlands;; 3 Department of Pathology, University Medical Center Groningen, Groningen, The Netherlands;; 4 Department of Surgery and Medical Imaging Center, University Medical Center Groningen, The Netherlands;; 5 TRACER EUROPE B.V. / AxelaRx, Groningen, The Netherlands;; 6 Center for Optical Diagnostics and Therapy, Department of Otorhinolaryngology and Head and Neck Surgery, Erasmus MC Cancer Institute, Rotterdam, the Netherlands.

**Keywords:** Structured roadmap, standardized fluorescence molecular endoscopy methodology, Barrett's esophagus, EMI-137 targeting c-Met.

## Abstract

Fluorescence molecular endoscopy (FME) is an emerging technique in the field of gastroenterology that holds potential to improve diagnosis and guide therapy, by serving as a 'red-flag' endoscopic imaging technique. Here, we investigated the safety, feasibility and optimal method of administration of EMI-137, targeting c-Met, during FME in Barrett's Esophagus (BE) and report several outcome parameters for early phase FME studies.

**Methods**: FME was performed in 15 Barrett's neoplasia patients. EMI-137 was administered to three cohorts of five patients: 0.13 mg/kg intravenously (IV); 0.09 mg/kg IV or topically at a dose of 200 μg/cm BE (n=1) or 100 μg/cm BE (n=4). Fluorescence was visualized *in vivo,* quantified *in vivo* using multi-diameter single-fiber reflectance, single-fiber fluorescence (MDSFR/SFF) spectroscopy and correlated to histopathology and immunohistochemistry. EMI-137 localization was assessed using fluorescence microscopy.

**Results**: FME using different IV and topical doses of EMI-137 appeared to be safe and correctly identified 16/18 lesions, although modest target-to-background ratios were observed (median range of 1.12-1.50). C-Met overexpression varied between lesions, while physiological expression in the stomach-type epithelium was observed. Microscopically, EMI-137 accumulated around the neoplastic cell membranes. We identified several outcome parameters important for the validation of EMI-137 for FME: 1) the optimal administration route; 2) optimal dose and safety; 3) *in vivo* FME contrast; 4) quantification of intrinsic fluorescence; 5) *ex vivo* correlation of fluorescence, histopathology and target expression; and 6) microscopic tracer distribution.

**Conclusions**: C-Met targeted FME using EMI-137 may not be the ideal combination to improve BE surveillance endoscopies, however the identified outcome parameters may serve as a valuable guidance for designing and performing future early phase clinical FME studies, independent of which fluorescent tracer is investigated.

## Introduction

Over the past decade, increasing knowledge on molecular and genetic alterations in gastrointestinal diseases along with technical improvements in endoscopy have led to the clinical translation of fluorescence molecular endoscopy (FME) 1. FME is an emerging technique in the field of gastroenterology that enables *real-time* visualization of disease-specific biomarkers, upregulated proteins or overexpressed receptors during endoscopy 2. Therefore, it holds great potential to improve diagnosis and guide therapy, by serving as a *'red-flag'* endoscopic imaging technique, which is demonstrated by several landmark papers that have been published on clinical phase I studies investigating the added value of FME for improved (pre)malignant lesion detection and prediction of treatment response 3-8.

There are numerous targeted fluorescent optical imaging agents in the early stage of clinical development. However, translation from bench-to-bedside can be highly challenging, in particular because of good manufacturing practice production of the imaging agent, safety and toxicity requirements, relatively high initial costs that are involved and insufficient resources or expertise for clinical translation 9. In addition, preclinical results that were obtained in mouse tumor xenograft or disease models are often not sufficiently representative for the human situation in terms of disease pathogenesis, pharmacokinetics, biodistribution and overall safety.

Despite these challenges, several targeted (near-infrared) fluorescent tracers have already been investigated for application in endoscopic imaging, for indications like colorectal polyp 4-6 and Barrett's neoplasia 7 detection or the evaluation of treatment response in inflammatory bowel disease 3 or locally advanced rectal cancer patients 8. Despite promising results, comparison between studies can be challenging, as different methodologies, analyses and outcome parameters have been used. This is mainly caused by the fact that fluorescence molecular imaging is not yet an established imaging modality and therefore currently no standards and guidelines exist that define FME-study objectives, protocols, outcome parameters or imaging specifications. Such standards and guidelines are especially important for the application of fluorescence molecular imaging in gastroenterology for reasons of obtaining uniformity in executing clinical studies, equipment and evaluation methods, as it is often challenging to correlate *in vivo* obtained fluorescence imaging results to *ex vivo* histopathology and immunohistochemistry, up to a microscopic level.

Therefore, to better validate a fluorescent tracer and to improve comparison between different FME study results, we have performed a c-MET targeted FME study in BE patients following a methodology that is based previous clinical studies in translating molecular fluorescence imaging into the clinic 5,7,8,10-13. The aim of such a methodology is to validate a fluorescent tracer in an early phase clinical study by investigating its safety, feasibility and most optimal dose in a well-informed way, without compromising data quality. Here, we demonstrate the practical application and the potential added value of such a methodology, by evaluating the safety, feasibility and optimal method of administration of EMI-137, a fluorescent peptide targeting the c-Met membrane receptor, during FME in patients with Barrett's neoplasia.

## Methods

### Study design

This study was a non-randomized, non-blinded, prospective, single-center safety, feasibility and dose-finding study that was conducted at the University Medical Center Groningen (UMCG). Fifteen patients with Barrett's Esophagus (BE) aged 18 years or above with a dysplastic lesion (TNM classification: ≤ cT1) scheduled to undergo an endoscopic mucosal resection (EMR) were included. Main exclusion criteria were any medical or psychiatric conditions that would compromise a patients' ability to give informed consent, pregnancy, breast feeding and lesions that were not suitable for EMR. All patients gave written informed consent. The study was approved by the medical ethics committee of the UMCG (METc 2016/595; 05-JAN-2017) and registered with ClinicalTrials.gov (Identifier: NCT03205501).

#### EMI-137, a fluorescent peptide targeting c-Met

C-Met is a transmembrane tyrosine kinase receptor that plays a role in tumor cell migration, invasion, proliferation and angiogenesis 14,15, which becomes overexpressed as the degree of dysplasia progresses in BE patients 16. The fluorescent tracer EMI-137 is a water-soluble 26-amino acid cyclic peptide conjugated to a Cy-5 derived fluorescent dye (peak excitation and emission: 653 nm and 675 nm) with a high affinity for the human c-Met receptor 6.

EMI-137 was administered either as an intravenous (IV) bolus injection ±2.5 h before the endoscopy procedure (4.8 mg/mL), or topically (100 or 200 μg/cm BE) during endoscopy using a spraying catheter. Further clarification on the IV dose and topical dose and incubation time is described in the 'Interim analysis' section below. Safety parameters such as heart rate, blood pressure, temperature and the presence of (serious) adverse events were monitored before and at regular intervals after EMI-137 administration.

### *In vivo* study procedures

All EMRs were performed according to standard clinical care based on high-definition, white-light endoscopy (HD-WLE). FME results did not influence clinical decision making. FME was performed using a fiber-bundle consisting of 30,000 coherent fibers (Schölly Fiberoptic GmbH, Denzlingen, Germany) coupled to the SurgVision Explorer Endoscope (SurgVision BV., Groningen, The Netherlands), a custom-build fluorescence endoscopy platform specifically developed to visualize EMI-137. The fiber-bundle has a field-of-view of 85°, can be inserted through the working channel of the clinical gastroscope and produces a white-light, fluorescence and overlay image for co-localization purposes. To prevent EMI-137 excitation by the Olympus xenon light-source (CLV-190, EVIS EXERA III, Olympus Corporation, Tokyo, Japan), a 650 nm short-pass filter (Chroma Technology Corp., Bellows Falls, VT, USA) was installed.

FME was performed directly after macroscopic identification of a location suspect for neoplasia by HD-WLE (further referred to as 'lesion'). A predetermined set of imaging parameters was used for all patients. Prior to topical administration, the esophagus was sprayed with acetylcysteine as an anti-mucolytic (4 mL, 100 mg/mL) and subsequently rinsed using 0.9% sodium chloride solution. After topical EMI-137 application, the esophagus was rinsed after a predetermined incubation time (see 'Interim analysis') using 0.9% sodium chloride solution, to remove any unbound EMI-137. FME was performed before (i.e. as a negative control) and after topical application of EMI-137. A qualitative assessment was performed on FME images (scaled per patient with minimum-maximum values) to define the fluorescence in the lesion as clearly increased, mildly increased or the same as the surrounding background fluorescence.

Subsequently, direct contact multi-diameter single-fiber reflectance, single-fiber fluorescence (MDSFR/SFF) spectroscopy measurements were acquired *in vivo* from the dysplastic lesion and surrounding squamous epithelium and BE if present. MDSFR/SFF spectroscopy enables the quantification of intrinsic fluorescence values by correcting for the tissue optical properties (i.e. scattering and absorption). First, the absorption coefficient and reduced scattering coefficient were measured at the EMI-137 excitation wavelength and over the emission band of the Cy-5 derived fluorescent dye (600-800 nm) through an optical fiber-bundle consisting of two fibers with different diameters. Subsequently, the raw fluorescence spectrum was measured. The intrinsic fluorescence values *(Q·μ^f^_a,x_)* were then calculated after the endoscopy procedure as described previously 17-19. All measurements were acquired in triplicate and median values with interquartile range were calculated per location.

In case additional lesions were detected using FME, biopsies were taken after MDSFR/SFF spectroscopy measurements to correlate fluorescence with histology.

### Interim analysis

An interim analysis was performed after the inclusion of five patients (0.13 mg/kg EMI-137 IV) to evaluate safety parameters, *in vivo* lesion identification using FME, MDSFR/SFF spectroscopy quantification data and *ex vivo* validation of EMI-137. In case high background fluorescence limited lesion detection, another five patients were included with a lowered dose (0.09 mg/kg EMI-137 IV), to decrease the amount of unbound circulating EMI-137. In parallel, in case fluorescence in the lesion appeared to be insufficient for adequate lesion detection, the dose could be increased (0.18 mg/kg EMI-137 IV).

If changing the IV dose still resulted in insufficient target-to-background ratios (TBR), the method of administration could be altered to topical application (200 μg/cm BE) to increase the mucosal tracer concentration, aiming to improve TBRs. The dose and incubation time (5 min) could be decreased (100 μg/cm BE) or increased (10 min) respectively, depending on *in vivo* results. In case sufficient TBRs were observed that allowed adequate *in vivo* lesion identification and discrimination from the non-dysplastic surrounding tissue 20, a total of a maximum of 20 patients could be included.

### *Ex vivo* validation

To confirm *in vivo* FME results, macroscopic and microscopic fluorescence imaging was performed *ex vivo* during all steps of pathological processing. After resection, a fluorescence image was acquired of each EMR specimen using the PEARL Trilogy Imaging System (LI-COR Biosciences, Lincoln, Nebraska, USA). A uniquely colored pin was placed at 12 o'clock to ensure identical orientation throughout pathological processing. Subsequently, the EMR-specimens were formalin fixated for 24 h and inked (blue ink on 3 o'clock side, black ink on 9 o'clock side). After histological slicing, the mucosal side of all tissue slices were imaged using the Odyssey CLx flatbed scanner (LI-COR Biosciences, Lincoln, Nebraska, USA). A hematoxylin and eosin (H/E), p53 and caldesmon staining were performed on 4 μm tissue sections for routine histopathological analysis according to standard clinical protocol of the UMCG.

c-Met immunohistochemistry was performed using the BenchMark ULTRA system (Ventana Medical Systems, Oro valley, Arizona, USA) using a SP44 rabbit monoclonal primary antibody directed against the membranous and cytoplasmic c-Met (Ventana Medical Systems). C-Met membrane expression was scored negative (0), weak (1+), moderate (2+) or strong (3+) by a board-certified pathologist blinded for the fluorescence imaging results. A score of moderate (2+) or strong (3+) was considered positive for c-Met membrane over-expression.

Fluorescence microscopy was performed to evaluate the EMI-137 distribution after a Hoechst nuclear staining (0.5 μg/ml Hoechst 33342, Thermo Fisher Scientific, Waltham, Massachusetts, USA), using a DM6000 fluorescence microscope (Leica Biosystems GmbH, Wetzlar, Germany) coupled to a DFC360FX camera. The following filter sets were used: FITC for autofluorescence, DAPI for the nuclei and Cy-5 for EMI-137 derived fluorescence (filter cubes A, I and Y5 respectively) with standardized settings for the Cy-5 channel.

*In vitro* experiments were performed to confirm binding specificity after topical administration of EMI-137 on two esophageal adenocarcinoma cell lines (OE-33, c-Met overexpression and FLO-1, negative c-Met expression). In short, c-MET expression was evaluated by immunohistochemistry and Western Blot analysis. EMI-137 binding localization was evaluated by fluorescence microscopy and cell membrane-binding affinity was confirmed by fluorescence-activated cell sorting analysis in a binding and blocking experiment (see supplementary methods).

### Statistical analyses

Normally distributed data was presented as mean values ± standard deviation (SD). A Student's t-test was used to test for significance for paired or independent data. Non-normally distributed data was presented as median values with interquartile range (IQR). A Mann-Whitney U test (independent data) or Wilcoxon signed rank test (paired data) was used to test for significance. P-values <0.05 were considered statistically significant. GraphPad Prism (version 8.0, GraphPad Software Inc, San Diego, California, USA) was used for data presentation and statistical tests.

## Results

### Patient characteristics and safety

Fifteen patients with a neoplastic lesion containing at least low-grade dysplasia were included in the study (Table 1). Five patients received 0.13 mg/kg EMI-137 IV, five patients received 0.09 mg/kg EMI-137 IV and five patients received a topical administration of EMI-137 (200 μg/cm BE (N=1) or 100 μg/cm BE (N=4)). IV administration of EMI-137 was performed 02:59±00:17 h (median ± IQR) before endoscopy. There were no (serious) adverse events related to any of the study procedures, nor were there clinically significant changes in vital parameters after the administration of EMI-137.

### Intravenous EMI-137 administration

**Figure 1:**
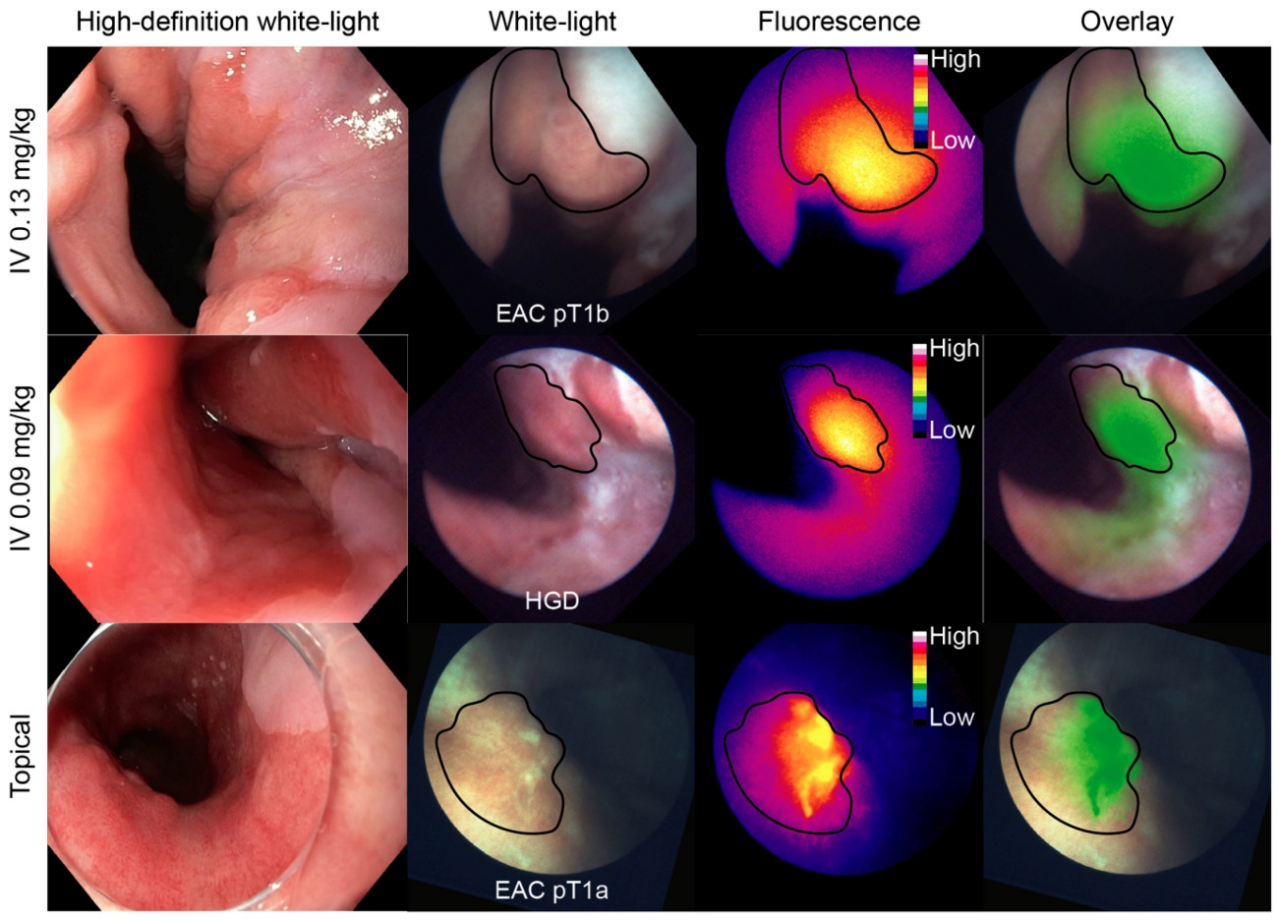
** Representative FME images per dose-cohort.** An esophageal adenocarcinoma (EAC), high-grade dysplastic (HGD) and EAC lesion from each cohort are displayed on each row respectively. The high-definition white-light image was acquired with the clinical video-endoscope, whereas the white-light, fluorescence and overlay images were acquired with the fiber. HD = high-definition; IV = intravenous.

Representative FME images of each dose-cohort are depicted in Figure 1. An overview of the *in vivo* study results of all individual patients is described in Table S1.

**Figure 2:**
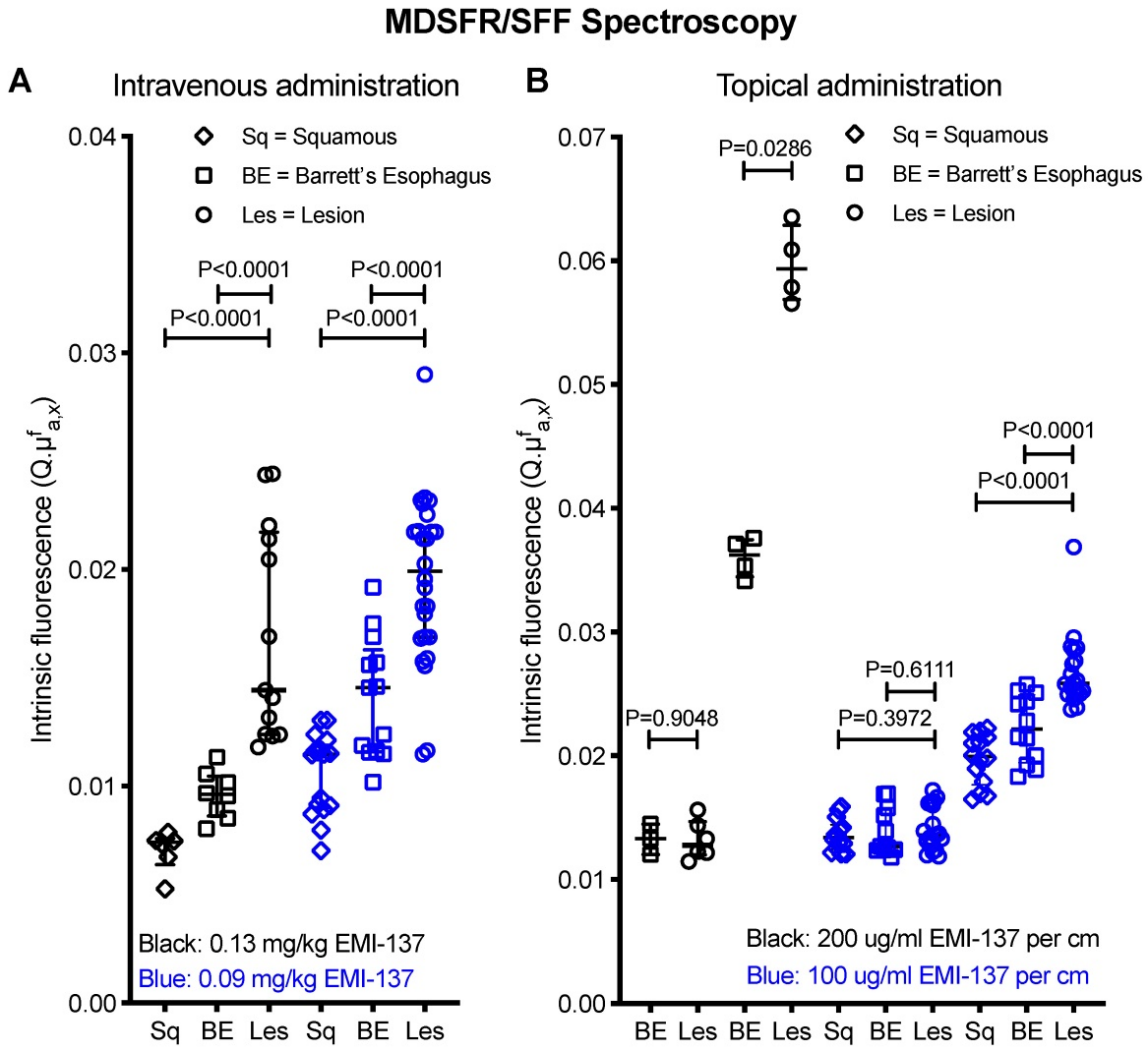
** Multi-diameter single-fiber reflectance, single-fiber fluorescence (MDSFR/SFF) spectroscopy data.** (A) Individual measurements of all intravenously administered patients grouped per cohort. The intrinsic fluorescence (Q·μ^f^_a,x_) is defined as the product of the fluorescence quantum yield of the Cy-5 derived fluorescence dye and it's absorption coefficient. Both in the 0.13 mg/kg and 0.09 mg/kg cohort, the lesion showed significantly increased fluorescence intensities compared to surrounding BE and squamous epithelium. *In vivo* MDSFR/SFF spectroscopy could not be performed in two patients of the 0.13 mg/kg cohort due to a malfunction of the device. (B) Individual measurements of all topically administered patients. Before topical application (T_0_), there was no statistically significant differences in FME and intrinsic fluorescence values between the lesion, BE and squamous epithelium, while after topical application (T_1_), there was a significant difference.

In the 0.13 mg/kg IV cohort, 3/4 lesions detected using HD-WLE (N=4 patients) were identified based on increased fluorescence intensities, although a modest contrast was observed with relatively high fluorescence in the surrounding BE and squamous epithelium. One patient was excluded from the *in vivo* analysis as FME could not be performed (Table S1). Quantified intrinsic fluorescence values of the lesions were significantly increased compared to surrounding BE (P<0.0001) and squamous epithelium (P<0.0001), with a median TBR of 1.50±0.65 and 2.43±0.64 respectively (Figure 2A). All lesions contained dysplasia/EAC on final histopathology. A moderate to strong c-Met membrane expression was observed in the lesions of all four patients that underwent FME (sensitivity 75%). Following our study methodology, we decided to lower the IV dose to 0.09 mg/kg to decrease background signals and improve TBRs.

In the 0.09 mg/kg IV cohort, 3/6 lesions detected using HD-WLE (N=5 patients) were identified during FME. Quantified intrinsic fluorescence values of the lesions were significantly increased compared to the surrounding BE (P<0.0001) and squamous epithelium (P<0.0001), with a median TBR of 1.27±0.15 and 1.71±0.29 respectively (Figure 2A). All three detected lesions had a moderate to strong c-Met membrane expression and proved dysplastic/EAC, whereas the three undetected lesions showed a weak c-Met expression, among which one benign lesion (sensitivity 100% and specificity 100% for the detection of c-Met expression levels using FME). However, lowering the EMI-137 dose did not decrease the background fluorescence compared to the 0.13 mg/kg IV cohort (Figure 2A).

**Figure 3:**
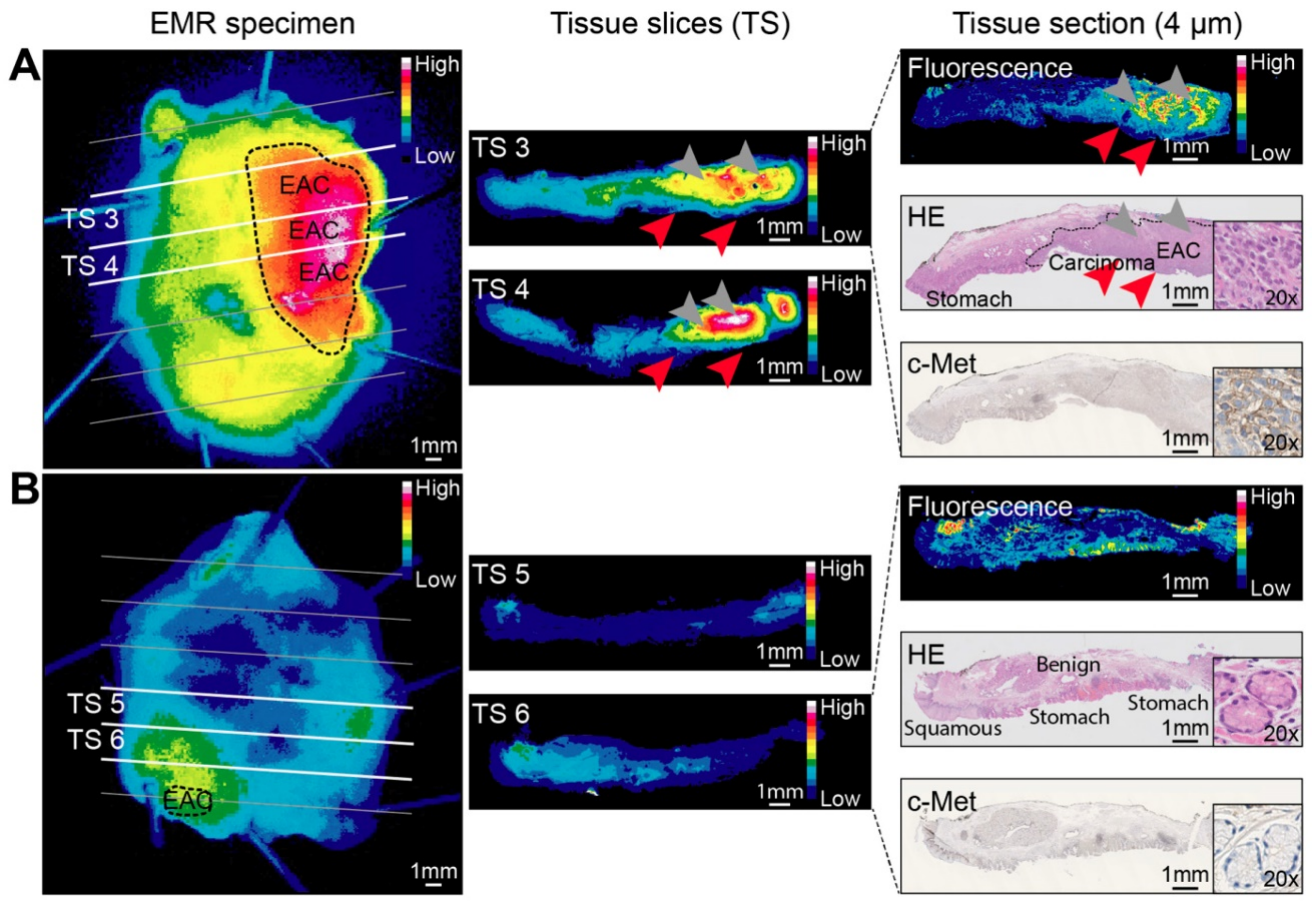
***Ex vivo* EMI-137 validation.** Representative fluorescence images acquired at every step during pathological processing from an esophageal adenocarcinoma (EAC) endoscopic mucosal resection (EMR) specimen (A) and a mainly benign EMR specimen consisting of BE and squamous epithelium (B) of the same patient. Images are scaled to each other per pathological processing step and can therefore be compared. A fluorescence image, hematoxylin and eosin (HE) staining and c-Met immunohistochemistry staining were performed on subsequent tissue sections for colocalization purposes. Arrowheads indicate relatively low fluorescence in the mucosa (red arrowheads) compared to the submucosa (gray arrowheads). Imaging of EMR specimens: PEARL Trilogy Imaging System; imaging of tissue slices and sections: Odyssey CLx flatbed scanner.

**Figure 4:**
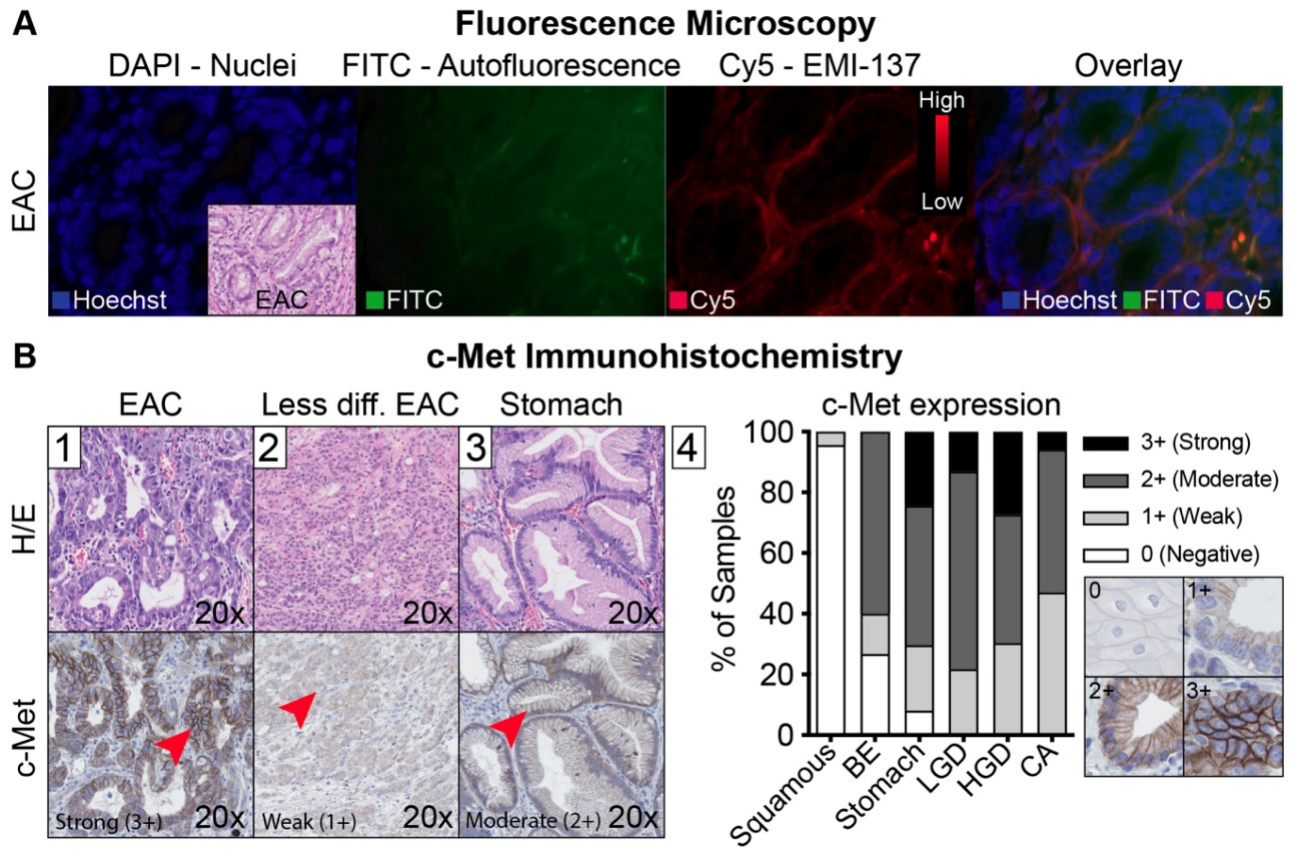
** Fluorescence microscopy and c-Met immunohistochemistry.** (A) Representative example of different fluorescence microscopy channels of one patient, showing increased fluorescence in the proximity of the adenocarcinoma cells. (B) Representative example of an esophageal adenocarcinoma (EAC) lesion with strong (3+) c-Met expression (B1), a less differentiated EAC lesion with a weak c-Met expression (B2) and foveolar epithelium of the stomach with moderate c-Met expression (B3). The graph shows overall c-Met expression in all 15 patients (B4).

*Ex vivo* correlation of fluorescence with histology showed a gross macroscopic colocalization of fluorescence intensities with dysplasia in 19/22 EMR specimens (Figure 3). High fluorescence intensities were observed in benign tissue in 2/22 EMR specimens and in one EMR specimen the lesion could not be discriminated based on fluorescence intensities. Fluorescence microscopy showed that EMI-137 was localized in the proximity of the dysplastic cells (Figure 4A). However, fluorescence imaging of tissue slices and sections showed fluorescence intensities were relatively low in the dysplastic mucosa compared to the submucosa (Figure 4A, red arrows).

### Topical EMI-137 administration

In order to achieve higher mucosal tracer concentrations compared to IV administration, we changed the administration route to topical administration of EMI-137. We first aimed to confirm topical EMI-137 binding specificity *in vitro*. Fluorescence microscopy showed fluorescence on the cell surface of c-Met positive EAC cells after topical administration (OE-33; Figure S1A), with a dose-dependent increase in EMI-137 membrane-binding using flow cytometry, while membrane-binding was blocked by addition of the non-fluorescent unlabeled peptide (AH111972; Figure S1B). In contrast, negligible fluorescence was observed on the c-Met negative EAC cells (FLO-1) using both methods (Figure S1).

Thereafter, EMI-137 was topically administered to another five patients. Autofluorescence levels were negligible compared to fluorescence intensities after topical administration (Figure S2). In the first patient of the topical cohort, both HD-WLE and FME detected one lesion (EAC) with a moderate c-Met expression, showing significantly increased fluorescence in the lesion compared to the surrounding squamous epithelium (P=0.0286), with a TBR of 1.64 (200 μg/cm BE EMI-137, Figure 2B). However, high fluorescence intensities saturated the fluorescence camera, even at the lowest exposure time and gain. Therefore, we decreased the dose to 100 μg/cm BE EMI-137 (N=4 patients). Subsequently, six out of seven lesions detected with HD-WLE showed increased fluorescence (Figure 1). Quantified intrinsic fluorescence values were significantly increased for the lesions versus surrounding BE (P<0.0001) and squamous tissue (P<0.0001), though with a moderate TBR of 1.12±0.11 and 1.33±0.12 respectively (Figure 2B). All lesions contained dysplasia/EAC except for one, which contained benign gastric epithelium, while all showed a moderate-to-high c-Met membrane expression (sensitivity 85.7%). In general, more speckled fluorescence patterns were observed after topical administration, probably as a result of a less homogenous tracer distribution. *Ex vivo* validation results showed that, after topical application, the majority of EMI-137 had been washed away during formalin fixation and paraffin embedding.

### Potential clinical added value of c-Met targeted FME

**Figure 5:**
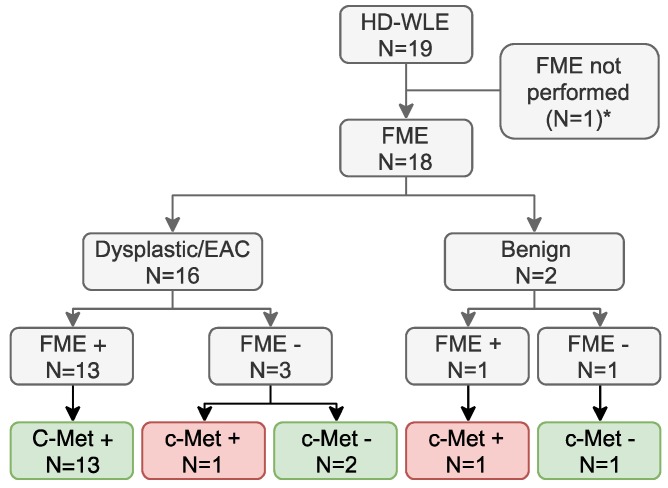
** Potential clinical benefit.** *FME could not be performed in one patient as the clinical gastroscope that should be coupled to the Olympus light source with the fluorescence filter installed, was unavailable. Of the 18 lesions in which fluorescence molecular endoscopy (FME) was performed, 16 were correctly identified, as 13 dysplastic or early stage esophageal adenocarcinoma (EAC) lesions with c-Met expression were fluorescence-positive and three lesions with a weak c-Met expression were fluorescence-negative (green boxes). One lesion was not identified using FME while expressing c-Met and one fluorescence- and c-Met-positive lesion was identified, despite not containing dysplasia (red boxes). HD-WLE = high-definition white-light endoscopy. *FME could not be performed due to logistical reasons in the 0.13 mg/kg cohort.

After validation of EMI-137 binding specificity, we assessed the potential clinical benefit of c-Met targeted FME, by evaluating its discriminative potential and the relation with c-Met expression levels. Using FME, 16 out of 18 lesions (89%) in which FME was performed were correctly identified (Figure 5). There were no additional lesions detected. MDSFR/SFF spectroscopy quantification showed increased fluorescence in the lesions compared to surrounding healthy tissue in all cohorts (Figure 2), although a modest contrast was observed with median TBRs ranging from 1.12 to 1.50. In addition, c-Met membrane overexpression was present in 14/17 lesions (82%; preliminary sensitivity 85.7%; specificity 100%) that contained dysplasia (Figure 4B-1, Table S1) and seemed to decrease in part of the less differentiated, invasive growing lesions (Figure 4B-2). Moreover, stomach-type epithelium also showed increased levels of c-Met membrane expression, which complicated lesion detection in the distal part of the esophagus, where the majority of neoplastic BE-lesions are located (Figure 4B-3/4).

### Lessons learned: proposal for outcome parameters in phase-I clinical FME studies

**Figure 6:**
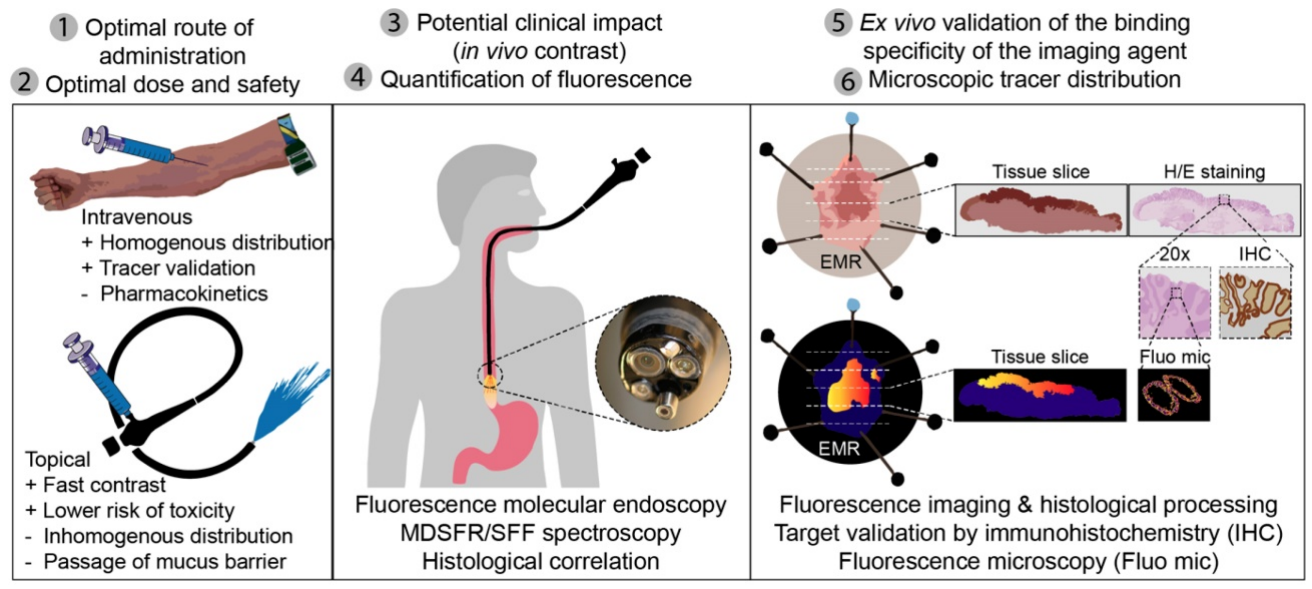
** Standardized fluorescence molecular endoscopy (FME) methodology.** Six outcome parameters were defined that are depicted here (1-6), with corresponding techniques or evaluation methods that should be performed in an early phase clinical study to evaluate a fluorescent tracer. MDRSFR/SFF = multi-diameter single-fiber reflectance, single-fiber fluorescence; EMR = endoscopic mucosal resection; H/E = hematoxylin and eosin.

Throughout the current study, we identified several outcome parameters that were important to provide reliable data when investigating a novel fluorescent tracer for FME, while limiting the number of patients exposed: 1) the optimal route of administration; 2) the optimal dose and safety; 3) the *in vivo* FME contrast to evaluate potential clinical added value; 4) the *in vivo* quantification of fluorescence with corrections for tissue optical properties; 5) the *ex vivo* validation by correlating fluorescence to histopathology and target expression levels; and 6) the evaluation of the microscopic tracer distribution (Figure 6). All of the above outcome parameters were reported in this phase-I clinical trial investigating FME using EMI-137.

## Discussion

FME is an emerging technique in the field of molecular fluorescence imaging, though standards and guidelines that define FME study designs, objectives and outcome parameters do not exist. We report the first results on FME using EMI-137, a fluorescent peptide targeting the c-Met membrane receptor, in patients with BE, using not only qualitative measurements such as *in vivo* FME or semi-quantification of fluorescence intensities on FME images, but also objective *in vivo* measurements using MDSFR/SFF spectroscopy. Using FME, 16 out of 18 lesions (89%) in which FME was performed were correctly identified related to histology and c-Met expression levels, although no additional lesions were detected. In addition, we have identified several outcome parameters that could guide the validation of a novel fluorescent tracer in phase-I FME studies.

The application of targeted fluorescent tracers has the potential to improve diagnostic and therapeutic endoscopy procedures by potentially guiding biopsies, improving lesion detection rates and restaging, thereby changing clinical decision making. In the emerging field of FME, a structured methodology is of particular importance considering all parameters that influence FME results, such as (fiber-based) fluorescence imaging resolution, different routes of administration, differences in tissue optical properties, imaging distance and illumination homogeneity, or the fact that piecemeal or part-by-part resections complicate *in vivo* to *ex vivo* correlation. Previously reported standards have been taken into account when identifying the outcome parameters that we propose 13,20-22. The clinical implementation of this methodology did not interfere with the clinical endoscopic and pathological workflow.

The optimal route of administration depends on the indication and organ of interest, but also on the pharmacokinetics, half-life and targeting ability of the imaging agent. For upper endoscopy, both IV and topical application are feasible. Topical application is less preferred for lower endoscopy, as this would require spraying of a relatively large surface and tracer binding is affected by the success of bowel preparation 23,24. A more homogenous tracer distribution can be achieved by IV administration, while spraying enables higher local (mucosal) tracer concentrations, with faster contrast and a substantially lower risk of toxicity (Figure 6) 7,23,24. For the purpose of validating a fluorescent tracer, IV tracer administration remains a crucial step for *ex vivo* validation and correlation, as topical tracer binding is affected by pathological processing 7. Although EMI-137 has favorable pharmacokinetics (t_1/2_ = 2 h 30 m) 6, for screening purposes in BE patients topical application would still be preferred from a clinical perspective.

When translating a novel fluorescent tracer into the clinic, the optimal dose should be established, which involves evaluation of the safety and imaging performance. To expose as little patients as possible, a traditional 3+3 dose-escalation design could be incorporated in the study design 25. In general, lower dosages are preferred due to a lower risk of side-effects and toxicity, provided adequate imaging performance is achieved. Here, we slightly deviated from this design, as the safety and feasibility of EMI-137 had been demonstrated in a previous study 6. However, we incorporated the option in our study design to change the dose based on *in vivo* results and to change the route of administration in order to achieve higher mucosal tracer concentrations. In addition, the dose and incubation time for topical application could be changed as well, which proved to be highly relevant when the fluorescence camera saturated using a dose of 200 μg/cm BE EMI-137.

The performance of a fluorescent tracer is influenced by differences in imaging distance, illumination homogeneity and tissue optical properties such as scattering and absorption. MDSFR/SFF spectroscopy is not subject to these factors, as it quantifies intrinsic fluorescence values by correcting for tissue optical properties through direct contact measurements. This provides quantitative values that can assist in evaluating the tracer distribution and support decision making on the optimal dose or dose-to-imaging interval 5,8. Here, we showed that MDSFR/SFF spectroscopy provides robust data with small confidence intervals, which assists in an objective comparison of the tracer distribution between dose-cohorts, or even between different FME studies.

This structured FME methodology is designed to fit both the endoscopy and pathology workflow, which allows a macroscopic-to-microscopic validation and correlation of *in* and *ex vivo* results. We specifically demonstrated EMI-137 accumulation in the proximity of the neoplastic cells by comparing EMI-137 derived fluorescence with autofluorescence using fluorescence microscopy, in addition to previous work 6. Moreover, fluorescence imaging during each pathological processing step contributed to the decision to change the route of administration, as limited EMI-137 accumulation was observed in the mucosa. Quantification of intrinsic fluorescence values confirmed that this indeed resulted in higher mucosal tracer concentrations compared to IV administration of EMI-137 (Figure 2).

The final objective was to evaluate whether c-Met targeted FME using EMI-137 has the potential to improve the neoplastic detection rate in BE-patients, which requires investigation of the previously discussed outcome parameters. Ultimately, to validate the clinical efficacy of a promising fluorescent tracer based on the phase-I study results, a subsequent phase-II diagnostic accuracy study would need to report important outcome parameters such as the sensitivity, specificity and negative and positive predictive value of the technique in a large cohort of BE patients. Here, we observed a variable c-Met membrane-expression between the lesions. In addition, the gastric foveolar epithelium also showed a physiological c-Met expression, while the majority of neoplastic lesions is located at the gastroesophageal junction. Secondly, TBRs for discriminating BE-tissue and the neoplasia of 1.12-1.50 may be insufficient for improved lesion detection in a general screening population.

In conclusion, although TBRs could perhaps be improved by further optimization of the tracer dose, we concluded that based on these results, c-Met targeted FME using EMI-137 may not be the ideal marker for application in BE surveillance endoscopies. In addition, we identified six important outcome parameters that fitted the clinical endoscopic and pathological workflow and may serve as a structured guidance for evaluation of a novel fluorescent tracer in early phase FME studies. The addition of quantification of intrinsic fluorescence values through correction for optical properties enables a more reliable and objective evaluation of the tracer distribution in different patients and between dose-cohorts. The proposed outcome parameters can serve as a valuable guideline for designing and performing future early phase clinical FME studies, independent of which fluorescent tracer is investigated.

## Supplementary Material

Supplementary methods, figures, and table.

## Figures and Tables

**Table 1:** ** Patient characteristics.** A total of 15 Barrett's Esophagus patients with a lesion containing at least low-grade dysplasia were included before endoscopic mucosal resection (EMR). A total of 19 lesions were detected using high-definition white-light endoscopy, two of which proved to be benign.

Characteristics	0.13 mg/kg IV (N=5*) No.	0.09 mg/kg IV (N=5) No.	Topical (N=5) No.
Sex			
Male	4	4	5
Female	1	1	0
Age (years)			
Median (range)	57 (54 - 75)	70 (60 - 72)	64 (49 - 76)
Histology Pre-EMR			
LGD	1	1	0
HGD	3	3	2
Adenocarcinoma	1	1	3
Histology EMR-specimen	(N=5 lesions)	(N=6 lesions)	(N=8 lesions)
Benign	0	1	1
LGD	1	0	3
HGD	1	4	1
Adenocarcinoma	3	1	3
Invasion depth			
m2 (lamina propria)	1	1	1
m3 (muscularis mucosae)	4	4	4
pTNM classification (8^th^ ed.)			
pT1a	3	5	3
pT1b	2	0	2

## Abbreviations

BE: Barrett's Esophagus
EMR: Endoscopic Mucosal Resection
FME: Fluorescence Molecular Endoscopy
HD-WLE: High-Definition White-Light Endoscopy
IV: Intravenous
IQR: Interquartile Range
MDSFR/SFF: Multi-Diameter Single-Fiber Reflectance, Single-Fiber Fluorescence
TBR: Target-to-Background Ratio
