# Supplementary material for: C-Met targeted fluorescence molecular endoscopy in Barrett's esophagus patients and identification of outcome parameters for phase-I studies
Source: Theranostics. 2020 Apr 6;10(12):5357–67. doi: 10.7150/thno.42224 (PMC7196285; doi:10.7150/thno.42224)
Supplement: Supplementary file 1 — Supplementary methods, figures, and table. [file thnov10p5357s1.pdf]

SUPPLEMENTARY MATERIAL (pages 1-5)

Supplemental Methods

EMI-137 binding specificity in vitro

In vitro EMI-137 binding experiments were performed to confirm binding specific after topical administration of EMI-137. Two esophageal adenocarcinoma cell lines were used: one with high overexpression of c-Met (OE-33) and one with a negligible c-Met expression (FLO-1). Cell culturing was performed in Gibco RPM I medium with 10% fetal calf serum (Bodinco BV, Alkmaar, The Netherlands). All experiments described in this *in vitro* experiment section were performed in triplicate. C-Met expression levels were confirmed by immunohistochemistry. Cells were incubated with a mouse-monoclonal c-Met primary antibody (sc-514148 clone D-4, 1:500, Santa Cruz Biotechnology) at room temperature for one h, with a secondary antibody (rabbit-anti-mouse-HRP, 1:100, DAKO, Santa Ana, CA, USA) at room temperature for 30 min and a tertiary antibody (goad-anti-rabbit-HRP, 1:100, DAKO). Subsequently, Western Blotting was performed to confirm c-Met expression on a protein level, as previously reported.[1] The same c-Met mouse-monoclonal antibody (sc-514148, 1:500) was used to incubate the blots overnight at 4 °C. A mouse anti-actin monoclonal antibody (Clone: C4, 1:10.000, MP biomedicals, Santa Ana, CA, USA) was used as a control.

Fluorescence microscopy was performed to evaluate EMI-137 binding specificity after topical application of EMI-137. Cells were incubated in a serum-free phenol-red free RPMI medium for seven h at 37 °C, washed with PBS at 4 °C and detached using a Gibco PBS-based enzyme-free cell dissociation buffer at room temperature. A total of 10 μg EMI-137 was used to incubate cells, or cells were incubated solely with the medium as a control for five min at 37 °C. A cytospin was used to concentrate cells after washing steps. Modified Kaisers glycerin combined with a Hoechst nucleus staining (0.5 μg/ml) was used for staining. A DM6000 fluorescence microscope coupled to a DFC360FX camera (Leica Microsystems, Wetzlat, Germany) was used on a 63 x magnification with fixed settings.

To confirm EMI-137 membrane binding, flowcytometry (i.e. fluorescence-activated cell sorting) analysis was performed. Cells were prepared as described previously.[25] Four different concentrations of EMI-137 (0.5, 5, 50 and 500 nM) and four different concentrations of the unlabeled peptide AH111972 (50 nM, 500 nM, 5 μM and 50 μM) were used to incubate cells to demonstrate binding affinity (EMI-137 alone) or blocking of the c-Met receptor (EMI-137 and AH111972 combined). Flowcytometry analysis were performed using the Accuri C6 flow cytometer (BD Biosciences, San Jose, CA, USA) with software version 1.0.264.21.

Supplementary Figures

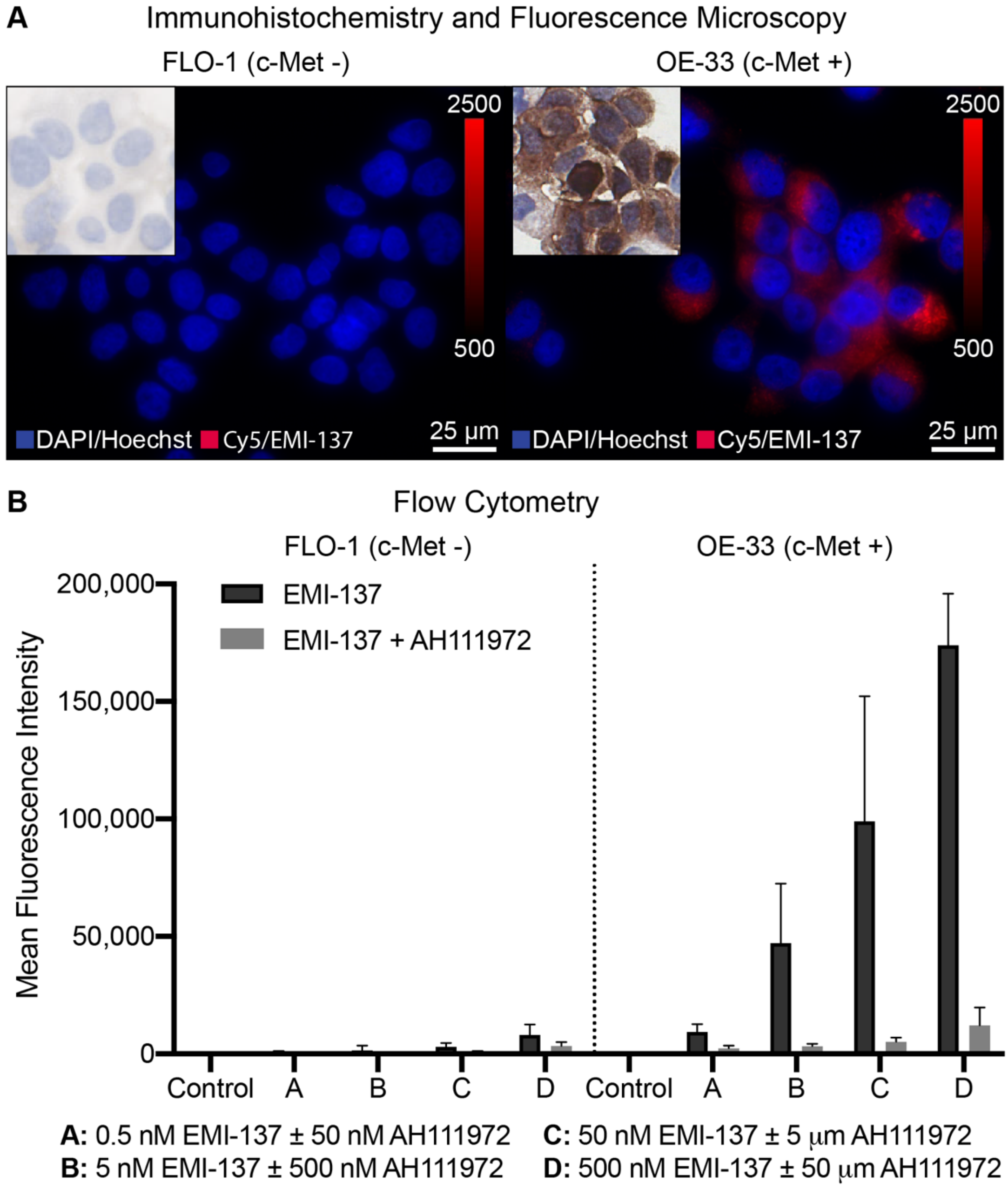

Figure S1. *In vitro* confirmation of EMI-137 binding specificity. (A) c-Met immunohistochemistry and fluorescence microscopy of the c-Met negative (FLO-1) and c-Met positive esophageal adenocarcinoma cell lines, showing negligible fluorescence and specific binding respectively. (B) Flow Cytometry experiment on both cell lines showing a dose-dependent specific membrane binding after topical administration of EMI-137, that was blocked by addition of the unlabeled c-Met specific peptide AH111972.

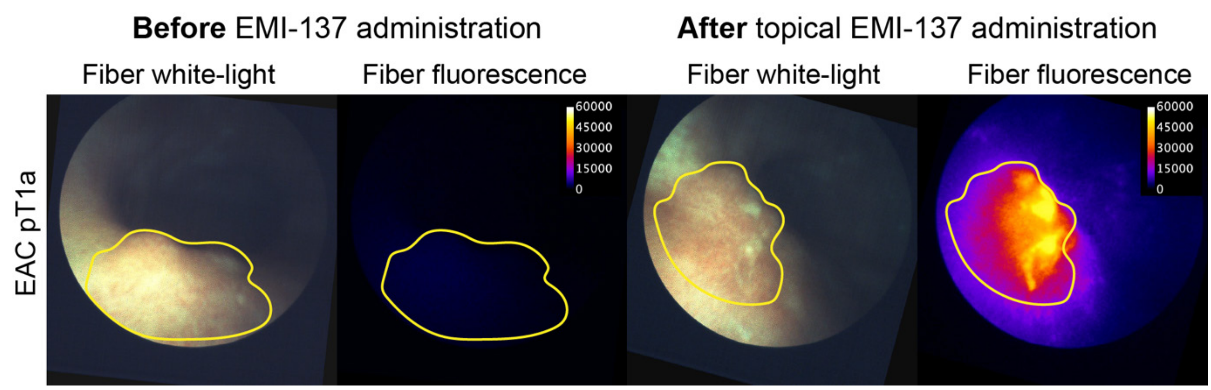

Figure S2. Representative autofluorescence image. White-light and fluorescence fiber image before topical application of EMI-137 (left) demonstrating the autofluorescence of the tissue, versus the same lesion after topical application of EMI-137, 5 min incubation time and rinsing using 0.9% sodium chloride solution (right). All fluorescence images are scaled equally and acquired of the same lesion using identical image acquisition parameters and can therefore be compared.

Supplementary Table

	Morphology	Histology	HD-WLE	FME	c-Met
Patient 1	Flat	HGD	Visible	Mildly increased	Moderate
Patient 2	Flat	Adenoca	Visible	Not applicable*	Weak
Patient 3	Flat	LGD	Visible	Same as background	Strong
Patient 4	Protruding	Adenoca	Visible	Increased	Moderate
Patient 5	Elevated	Adenoca	Visible	Increased	Moderate
Patient 6	Elevated	HGD	Visible	Increased	Moderate
Patient 7	Elevated	HGD	Visible	Increased	Moderate
Patient 8	Flat	HGD	Visible	Same as background	Weak
Patient 9 #1	Flat	HGD	Visible	Same as background	Weak
Patient 9 #2	Flat	Benign	Visible	Same as background	Weak
Patient 10	Protruding	Adenoca	Visible	Increased	Strong
Patient 11	Elevated	Adenoca	Visible	Increased	Moderate
Patient 12	Elevated	HGD	Visible	Increased	Moderate
Patient 13 #1	Protruding	Adenoca	Visible	Mildly increased	Strong
Patient 13 #2	Flat	LGD	Visible	Mildly increased	Moderate
Patient 14 #1	Flat	LGD	Visible	Same as background	Moderate
Patient 14 #2	Protruding	LGD	Visible	Mildly increased	Moderate
Patient 15 #1	Elevated	Adenoca	Visible	Increased	Strong
Patient 15 #2	Protruding	Benign	Visible	Mildly increased	Moderate

* FME could not be performed as the gastroscope that should be coupled to the Olympus white-light source with the fluorescence filter installed was unavailable.

Table S1. *In vivo* study results. HD-WLE = high-definition, white-light endoscopy; FME = fluorescence molecular endoscopy; HGD = high-grade dysplasia; LGD = low-grade dysplasia; Adenoca = adenocarcinoma.
